# The role of ADP-ribosylation in regulating DNA interstrand crosslink repair

**DOI:** 10.1242/jcs.193375

**Published:** 2016-10-15

**Authors:** Alasdair R. Gunn, Benito Banos-Pinero, Peggy Paschke, Luis Sanchez-Pulido, Antonio Ariza, Joseph Day, Mehera Emrich, David Leys, Chris P. Ponting, Ivan Ahel, Nicholas D. Lakin

**Affiliations:** 1 Department of Biochemistry, University of Oxford, South Parks Road, Oxford, OX1 3QU, UK; 2 Dunn School of Pathology, University of Oxford, South Parks Road, Oxford, OX1 3RE, UK; 3 MRC Human Genetics Unit, The MRC Institute of Genetics and Molecular Medicine, University of Edinburgh, Western General Hospital, Crewe Road, Edinburgh EH4 2XU, Scotland, UK; 4 Manchester Institute of Biotechnology, University of Manchester, Princess Street 131, Manchester, M1 7DN, UK

**Keywords:** *Dictyostelium*, ADP-ribosyltransferases, PARPs, Interstrand crosslink

## Abstract

ADP-ribosylation by ADP-ribosyltransferases (ARTs) has a well-established role in DNA strand break repair by promoting enrichment of repair factors at damage sites through ADP-ribose interaction domains. Here, we exploit the simple eukaryote *Dictyostelium* to uncover a role for ADP-ribosylation in regulating DNA interstrand crosslink repair and redundancy of this pathway with non-homologous end-joining (NHEJ). *In silico* searches were used to identify a protein that contains a permutated macrodomain (which we call aprataxin/APLF-and-PNKP-like protein; APL). Structural analysis reveals that this permutated macrodomain retains features associated with ADP-ribose interactions and that APL is capable of binding poly(ADP-ribose) through this macrodomain. APL is enriched in chromatin in response to cisplatin treatment, an agent that induces DNA interstrand crosslinks (ICLs). This is dependent on the macrodomain of APL and the ART Adprt2, indicating a role for ADP-ribosylation in the cellular response to cisplatin. Although *adprt2^−^* cells are sensitive to cisplatin, ADP-ribosylation is evident in these cells owing to redundant signalling by the double-strand break (DSB)-responsive ART Adprt1a, promoting NHEJ-mediated repair. These data implicate ADP-ribosylation in DNA ICL repair and identify that NHEJ can function to resolve this form of DNA damage in the absence of Adprt2.

## Abstract (precis)

**Summary:** Here, we identify a role for post-translational modification ADP-ribosylation in the response to DNA interstrand crosslinks in the model *Dictyostelium*.

## INTRODUCTION

ADP-ribosyltransferases (ARTs) catalyse the addition of single or poly(ADP-ribose) (PAR) moieties onto target proteins by mono-ADP ribosylation (MARylation) or poly-ADP ribosylation (PARylation), respectively (Gibson and Kraus, 2012; Vyas et al., 2014). ARTs are conserved in a wide variety of organisms, with 17 genes containing predicted ART domains being identified in humans (Hottiger et al., 2010). PARP1 and PARP2, the founder members of the ART family, in addition to PARP5a and PARP5b (also known as TNKS and TNKS2, respectively) are poly-ARTs. All other active ARTs catalyse MARylation (Vyas et al., 2014). ADP-ribosylation has been implicated in a wide variety of cellular processes including cell growth and differentiation, transcriptional regulation and programmed cell death (Hottiger et al., 2010; Messner and Hottiger, 2011; Quenet et al., 2009).

The best defined role of ARTs is in DNA repair, particularly of DNA strand breaks. PARP1 is recruited to and activated by DNA single-strand breaks (SSBs) and modifies a variety of substrates, including itself, proximal to the DNA lesion (Caldecott, 2008; Krishnakumar and Kraus, 2010). PARP1 is required for resolution of SSBs and disruption of its activity results in delayed repair and sensitivity to agents that induce base alkylation or DNA strand breaks (de Murcia et al., 1997; Ding et al., 1992; Fisher et al., 2007; Le Page et al., 2003; Masutani et al., 1999; Trucco et al., 1998). The finding that PARP2 catalyses residual PARylation in *Parp1^−/−^* cells led to the proposal that this ART also functions in SSB repair (Ame et al., 1999). Consistent with this model, *Parp2^−/−^* mice are sensitive to DNA damaging agents that induce strand breaks, in addition to displaying increased chromosome instability and delayed repair of damage following exposure to DNA alkylating agents (Menissier de Murcia et al., 2003; Schreiber et al., 2002). Although the relationship between PARP1 and PARP2 in regulating SSB repair is unclear, redundancy between these ARTs is implied by the embryonic lethality of *Parp1^−/−^Parp2^−/−^* mice (Menissier de Murcia et al., 2003).

ARTs are also crucial for resolution of DNA double-strand breaks (DSBs) by homologous recombination or non-homologous end-joining (NHEJ). PARP1 and PARP2 have been implicated in homologous recombination, particularly with reference to restart of stalled or damaged replication forks (Bryant et al., 2009; Sugimura et al., 2008; Yang et al., 2004). PARP1 is also required for alternative-NHEJ (A-NHEJ), an end-joining pathway activated in the absence of core NHEJ factors (Audebert et al., 2004; Brown et al., 2002; Robert et al., 2009; Wang et al., 2006). However, there are conflicting reports regarding the requirement for PARP1 in classic NHEJ (Luijsterburg et al., 2016; Yang et al., 2004). Instead, PARP3 PARylates targets at DSBs and promotes NHEJ by facilitating accumulation of repair factors such as APLF and Ku (a dimer of Ku80 and Ku70, also known as XRCC5 and XRCC6) at damage sites (Boehler et al., 2011; Couto et al., 2011; Loseva et al., 2010; Rulten et al., 2011).

A unifying theme of how ADP-ribosylation regulates resolution of DNA strand breaks, and possibly other varieties of DNA lesion, is through promoting the assembly of DNA repair and chromatin remodelling factors at damage sites. This is achieved through ADP-ribose interaction domains in these factors that interact with proteins that have been PARylated or MARylated at DNA lesions. The best characterised of these modules include a 20-amino-acid PAR-binding motif (PBM), PAR-binding zinc-finger (PBZ), macro and WWE domains (Gibson and Kraus, 2012). The PBM was the first ADP-ribose binding module to be identified and is present in a number of proteins, including several DNA damage response (DDR) factors (Gagne et al., 2008). PBZ domains are apparent in three vertebrate proteins, all of which have been implicated in the DDR, and are required to enrich CHFR and APLF at DNA damage sites (Ahel et al., 2008; Rulten et al., 2011). Although PBZ domains bind ADP-ribose polymers, macrodomains are more diverse in nature, binding a variety of ligands including PAR chains, mono-ADP-ribose units and O-acetyl ADP-ribose (Aravind, 2001; Han et al., 2011; Karras et al., 2005; Rack et al., 2016). Additionally, some macrodomains possess PAR and MAR-hydrolase activity, implicating these proteins in the removal of ADP-ribose moieties in order to regulate a variety of cellular processes (Barkauskaite et al., 2015; Jankevicius et al., 2013; Rosenthal et al., 2013; Sharifi et al., 2013; Slade et al., 2011). Despite this functional diversity, macrodomains uniformly adopt an α-β-α sandwich fold, with amino acid variations within a conserved binding pocket being responsible for the ligand-binding specificity or catalytic activity of each domain. Macrodomains have been identified in several DDR proteins and are required to recruit the chromatin remodelling factor ALC1 (also known as CHD1L) and the histone variant macroH2A1.1 to DNA damage (Ahel et al., 2009; Gottschalk et al., 2009; Timinszky et al., 2009).

Previously, we and others identified that the genetically tractable eukaryote *Dictyostelium discoideum* contains several DNA repair proteins that are absent or show limited conservation in other invertebrate model organisms (Block and Lees-Miller, 2005; Hsu et al., 2006; Hudson et al., 2005; Zhang et al., 2009). In this regard, several ARTs are apparent in the *Dictyostelium* genome (Pears et al., 2012), and, similar to in vertebrates, we find that two (Adprt1b and Adprt2) confer cellular resistance to SSBs (Couto et al., 2011)*.* A third ART (Adprt1a) is dispensable for SSB repair, but instead promotes NHEJ by facilitating accumulation of Ku at DSBs (Couto et al., 2011; Pears et al., 2012). Interestingly, the PBZ domain is unusually prevalent in *Dictyostelium*, with seven proteins containing this domain compared to three in vertebrates (Ahel et al., 2008). *Dictyostelium* Ku70 contains a PBZ domain, which is required for the enrichment of the protein in chromatin following DNA DSBs and to promote efficient NHEJ (Couto et al., 2011). Given that this motif is absent in vertebrate Ku70, these observations suggest that PBZ domains have been fused to a number of *Dictyostelium* DNA repair proteins during evolution. Therefore, the presence of other ADP-ribose interaction domains might act as a surrogate marker for new proteins involved in the DDR.

Although the role of ARTs in DNA strand break repair is well established, whether these enzymes regulate other repair processes remains unclear. Here, we exploit the increased frequency of ADP-ribose interaction motifs in *Dictyostelium* to uncover a role for ADP-ribosylation in regulating repair of DNA damage inflicted by cisplatin, an agent that induces DNA interstrand crosslinks (ICLs). Through an *in silico* approach to identify new macrodomain-containing proteins in this organism, we identify a protein containing regions of similarity to aprataxin, APLF and PNKP that we call APL (aprataxin/APLF-and-PNKP-like protein). APL is recruited to DNA damage induced by cisplatin in a manner that is dependent on its macrodomain. Consistent with these observations, we report that ADP-ribosylation is induced in response to cisplatin, and that ARTs are required for tolerance to DNA damage induced by this agent. Finally, we exploit the genetic tractability of *Dictyostelium* to uncover a new level of redundancy between ARTs and the NHEJ pathway in allowing cells to tolerate cisplatin exposure.

## RESULTS

### Identification of new *Dictyostelium* macrodomain-containing proteins

**Fig. 1.**
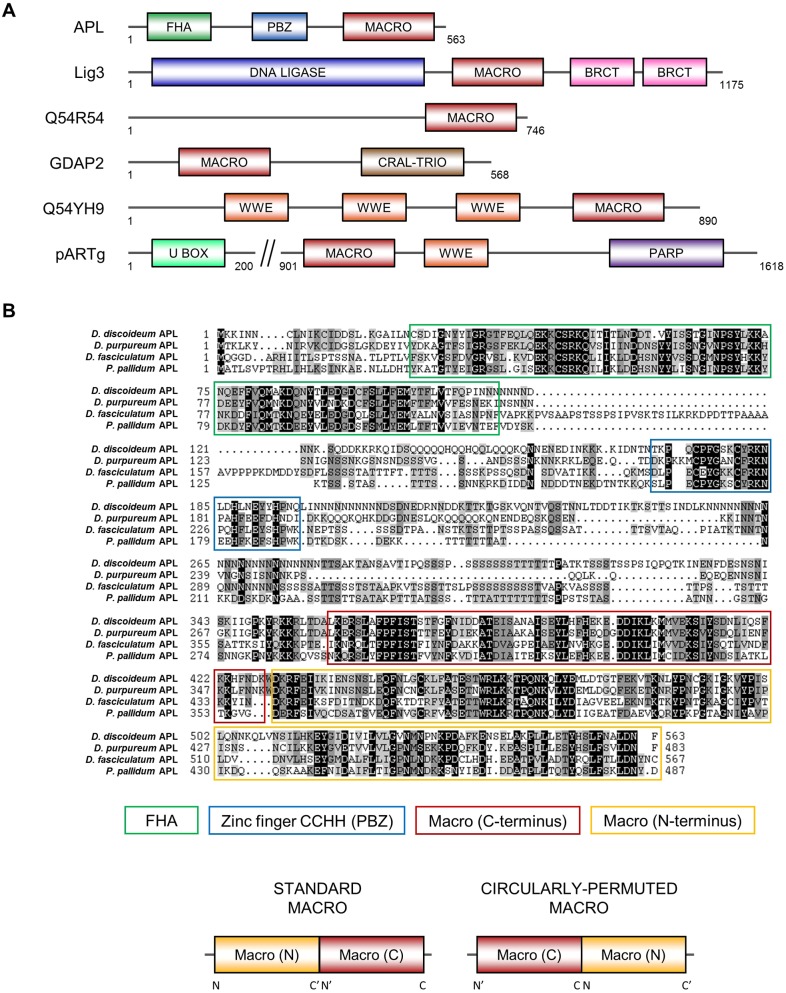
**The *Dictyostelium* protein APL contains a circularly permuted macrodomain.** (A) Domain structures of the *Dictyostelium* macrodomain (MACRO)-containing proteins identified through *in silico* searches. The macrodomains in GDAP2, Q54YH9 (UniProt ID) and pARTg were previously annotated. Domain abbreviations: FHA, forkhead-associated; PBZ, zinc finger CCHH-type; BRCT, BRCA1 C-terminus; CRAL-TRIO, CRAL-TRIO lipid binding domain; U-BOX, U-box domain; PARP, PARP catalytic domain. (B) Multiple sequence alignment of APL from different dictyostelids, highlighting the domain conservation between the proteins. This alignment shows the conservation of a circularly permutated macrodomain, which is illustrated relative to the standard macrodomain. Circular permutation is likely to have arisen from the duplication of the C- and N-terminal regions of successive macrodomains. For this to occur, macrodomains would need to occur in tandem in the progenitor protein, as indeed they do in many extant macrodomain-containing proteins. In the circularly permutated macrodomain, the N- and C-termini of the standard macrodomain lie in the middle of the domain sequence.

Previous bioinformatics analysis has indicated that there is an increased frequency of PBZ-domain-containing proteins in *Dictyostelium* relative to humans (Ahel et al., 2008). The majority of these proteins are orthologues of vertebrate factors previously implicated in the DDR. We hypothesised that this might also be the case for other ADP-ribose-binding modules, and thus that the presence of these domains could serve as surrogate markers for new DDR proteins. Although ADP-ribose-binding macrodomains have been identified in human DNA repair proteins (Ahel et al., 2009; Nicolae et al., 2015), these modules are evolutionarily diverse and exhibit a high level of primary sequence divergence that hinders their identification and annotation (Rack et al., 2016). Therefore, we sought to identify previously unannotated *Dictyostelium* macrodomain-containing proteins in the hope that this would uncover new proteins with a role in the DDR. Accordingly, we performed a genome-wide search using the primary sequence of known human macrodomains as the starting point for homology detection and subsequent generation of profile hidden Markov models (profile-HMMs) (Eddy, 1998). Profile-HMMs are mathematical constructs that incorporate the amino acid variation at each position in a multiple sequence alignment of a domain family, thereby providing more sensitivity than performing homology searches with an input of a single sequence. Given we sought to identify ADP-ribose-binding domains, we used the sequence of macrodomains known to interact with ADP-ribose in our searches, such as that found in ALC1 (Ahel et al., 2009). This approach yielded six *Dictyostelium* proteins with macrodomains, three of which had not been previously annotated in protein databases (Fig. 1A).

**Fig. 2.**
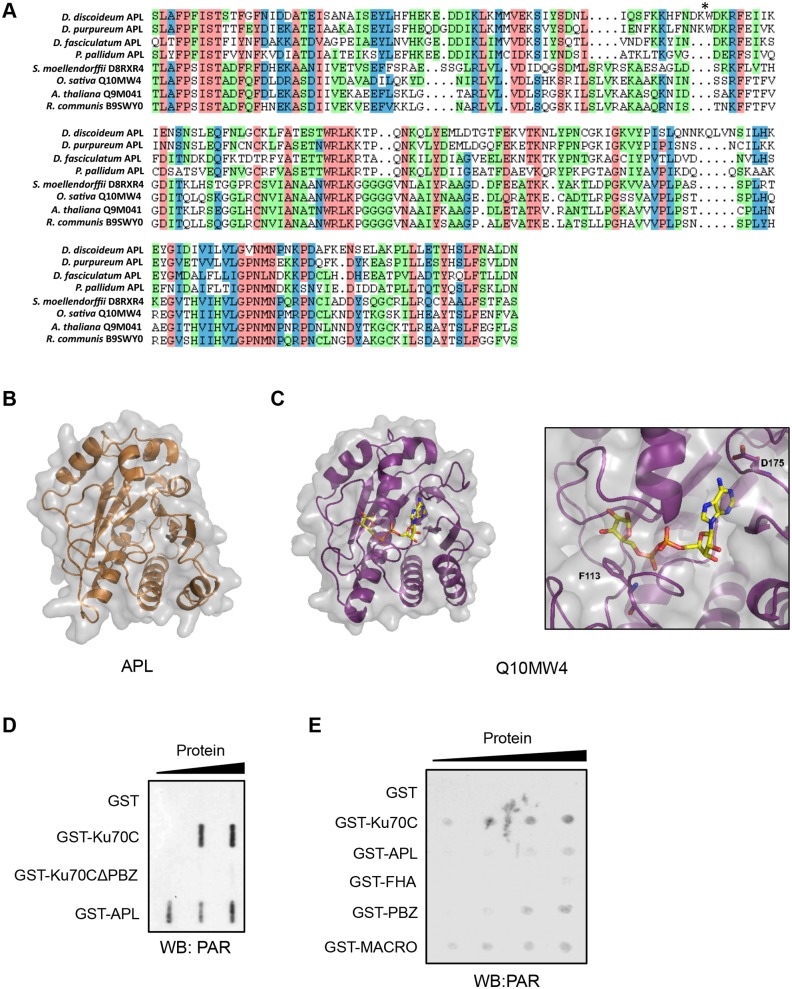
**The macrodomain of APL binds to PAR *in vitro.*** (A) Multiple sequence alignment of permutated macrodomains identified from a BLAST database search with the macrodomain sequence of APL. Proteins are identified as either dictyostelid orthologs of APL, or by UniProt accession number. The permutation site is marked by an asterisk. (B) Crystal structure of the permuted macrodomain from *Dictyostelium* APL. (C) Crystal structure of the permuted macrodomain from *O. sativa* Q10MW4 in a complex with ADP-ribose. A focus on the binding pocket of the permutated macrodomain indicates amino acids predicted to facilitate ADP-ribose binding and specificity: F113 and D175. (D) *In vitro* PAR-binding activity of APL. The indicated recombinant GST-tagged proteins were slot-blotted onto a nitrocellulose membrane in increasing concentrations, prior to incubation of the membrane with PAR polymers. Detection of bound PAR was performed by western blotting with an anti-PAR antibody. (E) *In vitro* PAR-binding activity of the isolated domains of *Dictyostelium* APL. The indicated GST-tagged proteins were dot-blotted onto a nitrocellulose membrane, prior to incubation with PAR polymers and detection by western blotting as in D.

One of the unannotated macrodomain containing proteins is the *Dictyostelium* orthologue of DNA ligase III. Given that vertebrate DNA ligase III does not contain a macrodomain, this supports our hypothesis that these modules can act as markers for DNA repair proteins in *Dictyostelium*. A further protein identified in this screen (UniProt Q54B72, gene DDB_G0293866) contained a macrodomain at its C-terminus and a central PBZ domain with predicted PAR-binding activity, in addition to an N-terminal FHA-like domain similar to those found in the human DNA repair proteins aprataxin, APLF and PNKP (Fig. 1A,B) (Ali et al., 2009; Chappell et al., 2002; Clements et al., 2004; Iles et al., 2007). Given the similarity of this protein to aprataxin, APLF and PNKP, we called this factor aprataxin/APLF-and-PNKP-like protein (APL). Interestingly, the N- and C-termini of the APL macrodomain align with the C- and N-termini of human macrodomains, respectively (Fig. S1), indicating it has undergone a circular permutation during evolution (Ponting and Russell, 1995). This circularly permuted macrodomain was found to be present in orthologues of APL in other dictyostelids (Fig. 1B). Such a permutation involving gross rearrangements of the primary sequence could result in severe tertiary structural alterations, impacting on the functionality of the macrodomain. Therefore, we assessed whether or not this circular permutation has affected the functional structure of the domain. First, we investigated whether this permutation was a unique event in dictyostelids or one that was evolutionarily conserved across other species, thereby providing evidence that it might be required for a biological function. A BLAST database search with the macrodomain sequence of APL identifies the permuted macrodomain in a small number of other organisms, including the plants *Arabidopsis thaliana* and *Oryza sativa*. These permutated domains show a high level of primary sequence conservation with that found in APL (Fig. 2A), indicating that the same permuted macrodomain is present in several diverse species and implying that it is functionally important.

In order to assess whether these permuted macrodomains retain important structural characteristics, we solved the X-ray crystal structures of the isolated permutated macrodomains found in *Dictyostelium* APL and *O. sativa* Q10MW4 (Fig. 2B,C; Table S1). A selenomethionine-substituted protein of the *Dictyostelium* macrodomain was used to collect single wavelength anomalous diffraction X-ray data, which was phased with AUTOSOL (Terwilliger et al., 2009). Subsequently, X-ray data from the *O. sativa* macrodomain was solved through molecular replacement with PHASER (Storoni et al., 2004) by using the *Dictyostelium* macrodomain structure as the search model. Previously solved structures of classical macro domains indicate that they consist of a non-parallel β-sheet core flanked by α-helices, with a cleft forming the binding pocket for ADP-ribose (Rack et al., 2016). These structural features are conserved in the permuted macrodomain found in *Dictyostelium* APL (Fig. 2B), indicating that the permutation does not drastically alter the structure of the domain. We were able to obtain the *O. sativa* macrodomain in a complex with ADP-ribose (Fig. 2C), further confirming that the canonical mode of interaction with ADP-ribose is also retained. For example, the acidic amino acids D175 (E439 in *Dictyostelium*) that forms hydrogen bonds with the ADP-ribose ligand, and the aromatic F113 that forms the binding pocket for the distal ribose unit are found in *O. sativa* Q10MW4 and canonical macrodomains, suggesting that these amino acids will perform the same functions in most macrodomains (Ahel et al., 2009).

To more formally assess whether APL is indeed an ADP-ribose-binding protein, and which domains of this protein are responsible, we expressed and purified a GST-tagged form of APL (GST–APL) and tested its ability to bind PAR polymers *in vitro* utilising a slot blot assay. Consistent with a previous report (Couto et al., 2011), a C-terminal region of Ku70 displayed PAR-binding activity in this assay in a manner that was dependent on its PBZ domain (Fig. 2D). Importantly, GST–APL also binds PAR, indicating that this protein is able to interact with ADP-ribose polymers *in vitro*. Given APL contains both PBZ and macrodomains capable of interacting with ADP-ribose polymers, we next determined which domains of APL are responsible for PAR-binding by assessing their ability to interact with PAR *in vitro*. Although the FHA domain of APL exhibited limited ability to interact with PAR *in vitro*, both the macro and PBZ domains of APL interacted with ADP-ribose polymers (Fig. 2E). Taken together, these data indicate that APL is indeed able to interact with PAR *in vitro* and that both the PBZ and macrodomains of the protein are able to perform this function.

### The macrodomain is required to enrich APL on chromatin in response to cisplatin-induced DNA damage

**Fig. 3.**
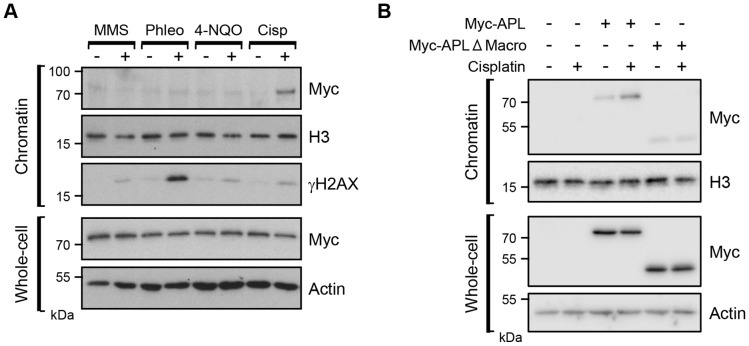
**APL is enriched on chromatin following cellular exposure to cisplatin in a manner dependent on its macrodomain.** (A) *Dictyostelium apl^−^* cells expressing Myc–APL were treated with the indicated DNA-damaging agents and chromatin or whole-cell extracts prepared. Western blotting was performed with the indicated antibodies. (B) *Dictyostelium apl^−^* cells expressing full-length Myc-tagged APL (Myc–APL), or a form of APL with its macrodomain deleted (Myc–APL-ΔMacro), were exposed to cisplatin alongside cells transfected with an empty vector. Chromatin fractions and whole-cell extracts were prepared and western blotting performed with the indicated antibodies. The image shown is representative of four independent experiments.

The N-terminal FHA domain of APL is most similar to those that facilitate the interaction of aprataxin, APLF and PNKP with the DNA repair proteins XRCC1 and XRCC4 (Ali et al., 2009; Chappell et al., 2002; Clements et al., 2004; Iles et al., 2007). Taken together with the presence of a PBZ domain, a motif present in proteins that function in the DDR, this suggests a role for APL in DNA repair. To investigate this, we generated a strain disrupted in the *apl* gene (Fig. S2) and assessed whether recombinant Myc-tagged APL expressed in these cells was enriched in chromatin following exposure to a specific form of genotoxic stress. No substantial enrichment of Myc–APL was observed in chromatin fractions prepared from cells exposed to agents that induce base damage (methyl methanesulphonate; MMS), DNA DSBs (phleomycin) or bulky adducts repaired by nucleotide excision repair (4-nitroquinoline-1-oxide; 4-NQO), despite the induction of DNA damage under these conditions, as judged by elevated γH2AX (Fig. 3A). Strikingly, however, we observed elevated levels of Myc–APL in chromatin fractions following exposure to the DNA ICL-inducing agent cisplatin, implicating APL in the response to DNA damage inflicted by this agent.

To determine whether the macrodomain of APL is required for this function, we generated a Myc-tagged form of APL with the macrodomain deleted and assessed its ability to assemble in chromatin following DNA damage. As observed previously, wild-type APL is effectively enriched in chromatin following exposure of cells to cisplatin (Fig. 3B). Strikingly, deletion of the macrodomain almost totally eliminates enrichment of APL in chromatin in response to cisplatin. Taken together, these data indicate APL as a new sensor for cisplatin-induced DNA damage and that the macrodomain of this protein is required for this function.

### Nuclear ADP-ribosylation is induced following cisplatin treatment

**Fig. 4.**
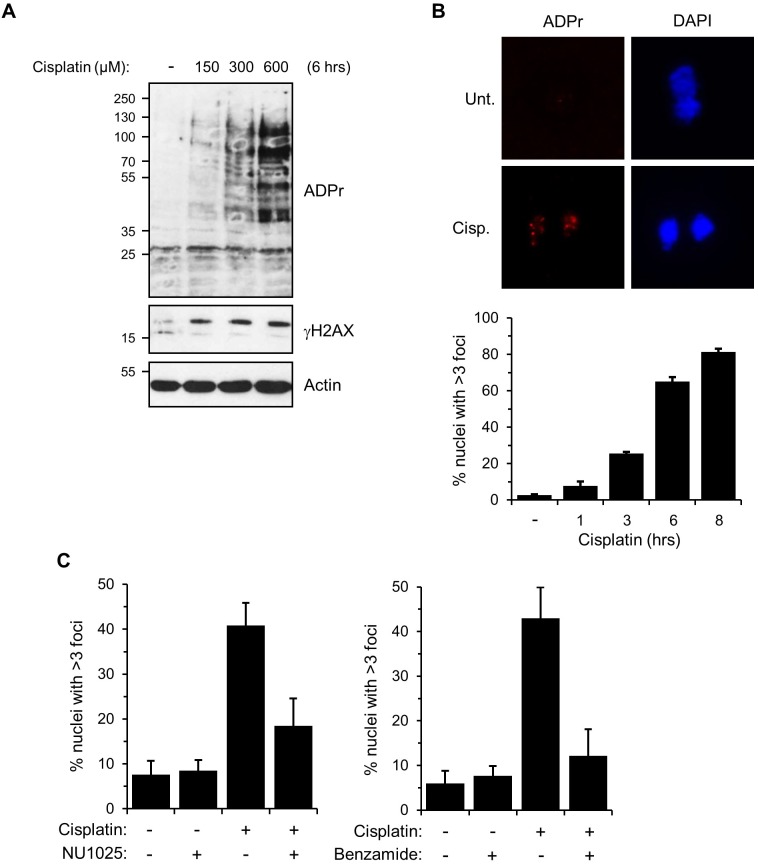
**Nuclear ADP-ribosylation is induced following cisplatin treatment.** (A) *Dictyostelium* Ax2 cells were exposed to the indicated concentrations of cisplatin for 6 h prior to preparation of whole-cell extracts. Western blotting was performed with the indicated antibodies. (B) Ax2 cells were treated with 300 μM cisplatin for 6 h, prior to nuclear extraction and staining with the indicated reagents for immunofluorescence. Images are representative of 250 nuclei. Error bars represent the s.e.m. from three independent experiments. ADPr, ADP-ribosylation. (C) Quantification of the effect of treatment with the PARP inhibitors NU1025 and benzamide on nuclear ADPr foci formation resulting from cisplatin exposure. Error bars represent the s.e.m. from four independent experiments.

Although the role of ARTs in SSB and DSB repair is well established, whether these enzymes are required for repair of other varieties of DNA lesions, such as DNA ICLs, is unknown. Our data indicating that the macrodomain of APL interacts with ADP-ribose polymers *in vitro*, taken together with the requirement for this domain for APL to be enriched in chromatin following exposure of cells to cisplatin, implicates ADP-ribosylation in the cellular response to DNA ICLs. To assess this possibility, we investigated whether ADP-ribosylation is induced in response to cisplatin. Ax2 cells were exposed to increasing doses of cisplatin, and ADP-ribosylation in whole-cell extracts was assessed by western blotting with reagents that detect both MARylation and PARylation (Fig. 4A). We observed a dose-dependent increase in ADP-ribosylated proteins in cells, indicating that cisplatin does induce cellular ADP-ribosylation. Moreover, consistent with ADP-ribosylation being induced at DNA damage sites, we observe the formation of ADP-ribosylation nuclear foci in a time-dependent manner, with 81% of cells containing greater than three foci after 8 h of cisplatin treatment (Fig. 4B). Pre-treatment of Ax2 cells with PARP inhibitors that inhibit ADP-ribosylation in *Dictyostelium* (Couto et al., 2011) significantly reduces the number of nuclei exhibiting ADP-ribosylation (Fig. 4C), indicating *Dictyostelium* ARTs are activated in response to cisplatin treatment.

### Adprt1a-mediated NHEJ is required for tolerance of cisplatin-induced DNA damage in the absence of Adprt2

**Fig. 5.**
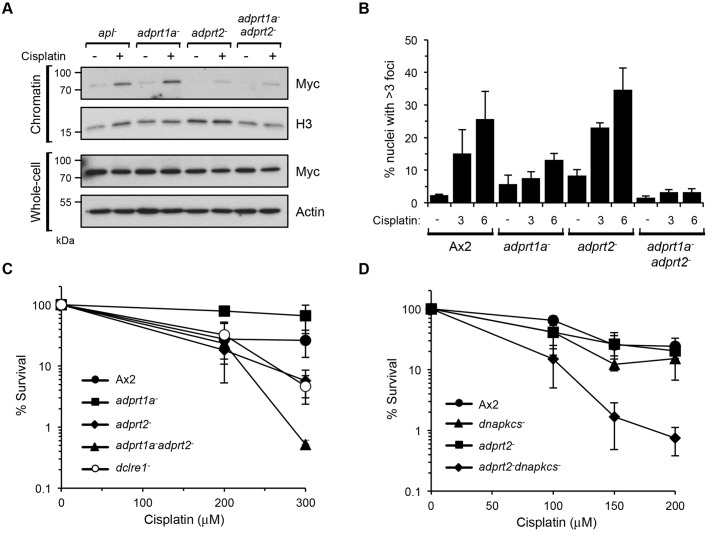
**NHEJ provides resistance to interstrand crosslinks in the absence of Adprt2.** (A) *Dictyostelium adprt1a^−^*, *adprt2^−^* and *adprt1a^−^adprt2^−^* cells expressing Myc–APL were left untreated or exposed to 300 μM cisplatin for 5 h. Chromatin and whole-cell extracts were prepared and western blotting performed with the indicated antibodies. The image shown is representative of three independent experiments. (B) Ax2, *adprt1a^−^*, *adprt2^−^*, *adprt1a^−^adprt2^−^* cells were exposed to 300 μM cisplatin for 6 h, prior to nuclear extraction and staining for immunofluorescence. Error bars represent the s.e.m. from three independent experiments. (C) Ax2, *adprt1a^−^*, *adprt2^−^*, *adprt1a^−^adprt2^−^* cells were assessed for survival after treatment with the indicated concentrations of cisplatin. Error bars represent the s.e.m. from three independent experiments. (D) Ax2, *dnapkcs^−^*, *adprt2^−^* and *adprt2^−^dnapkcs^−^* cells were assessed for survival after treatment with the indicated concentrations of cisplatin. Error bars represent the s.e.m. from four independent experiments.

We wished to identify the ARTs responsible for cisplatin-induced ADP-ribosylation. Similar to in humans, two *Dictyostelium* ARTs (Adprt1b and Adprt2) are required for tolerance of cells to DNA SSBs, whereas a third ART (Adprt1a) is required to promote NHEJ of DNA DSBs (Couto et al., 2013, 2011). We therefore considered whether any of these ARTs are similarly required for the cellular response to cisplatin. APL enrichment in chromatin following cisplatin exposure is dependent on the macrodomain of the protein (Fig. 3B), suggesting that ART-mediated ADP-ribosylation regulates this process. Therefore, we initially tested whether cisplatin-induced enrichment of APL in chromatin is dependent on Adprt1a or Adprt2. Accumulation of Myc–APL in chromatin following exposure of *adprt1a^−^* cells to cisplatin remained largely intact relative to *apl^−^* cells (Fig. 5A; Fig. S3). Despite basal levels of APL in chromatin being less in *adprt2^−^* and *adprt1a^−^adprt2^−^* cells in the absence of cisplatin (Fig. 5A), these strains displayed a significant reduction in cisplatin-induced enrichment of APL in chromatin (Fig. 5A and Fig. S3), indicating that Adprt2 is required to enrich and/or retain APL at DNA lesions induced by cisplatin.

Next, we assessed whether the requirement for Adprt2 to assemble APL in chromatin following exposure to cisplatin is reflected in the ability of these cells to induce nuclear ADP-ribosylation following DNA damage. Compared to parental Ax2 cells, a slight but not significant decrease in ADP-ribosylation was apparent in *adprt1a^−^* cells following exposure to cisplatin. Surprisingly, despite a reduction in macrodomain-dependent accumulation of APL in chromatin following exposure of *adprt2^−^* cells to cisplatin (Figs 3B and 5A), substantial nuclear ADP-ribosylation was apparent in these cells (Fig. 5B). However, this is dramatically reduced in the *adprt1a^−^adprt2^−^* strain, indicating that although Adprt2 is required to signal cisplatin-induced DNA damage and promote assembly of APL in chromatin, in the absence of this ART, Adprt1a can act as a signal of this variety of DNA damage. Further evidence for this redundancy is provided by analysing the tolerance of *adprt1a^−^*, *adprt2^−^* and *adprt1a^−^adprt2^−^* strains to cisplatin treatment. Consistent with a lack of requirement for Adprt1a in producing ADP-ribosylation foci in response to cisplatin, *adprt1a^−^* cells were no more sensitive to this genotoxin that parental Ax2 cells. However, the *adprt2^−^* strain was sensitive to cisplatin to a similar degree to cells disrupted in *dclre1*, the *Dictyostelium* orthologue of SNM1A (also known as DCLRE1A), a gene required for tolerance to ICLs in a variety of organisms (Dronkert et al., 2000; Henriques and Moustacchi, 1980; Wang et al., 2011). Interestingly, disruption of *adprt1a* in combination with *adprt2* further sensitised cells to cisplatin relative to the *adprt2^−^* strain. Assessed collectively, these data suggest that at least two redundant ART-dependent pathways operate in *Dictyostelium* in response to cisplatin: one mediated by Adprt2 and involving APL, and a secondary pathway dependent on Adprt1a.

Our previous work indicates that loss of Adprt2 results in increased DNA DSBs following exposure of cells to DNA alkylating agents and that this is subsequently signalled by Adprt1a to promote NHEJ and cell survival in response to these genotoxins (Couto et al., 2013). Given the redundancy between Adprt1a and Adprt2 in signalling cisplatin-induced DNA damage, we considered whether similar mechanisms are being employed in response to this variety of DNA damage. To test this hypothesis, we assessed the survival of NHEJ-deficient *dnapkcs^−^* cells, *adprt2^−^* cells and *adprt2^−^dnapkcs^−^* cells to cisplatin treatment. Consistent with previous data, *adprt2^−^* cells were sensitive to cisplatin treatment. Strikingly, whereas disruption of the *dnapkcs* gene alone had a minimal impact on the sensitivity of Ax2 cells to cisplatin, the *adprt2^−^dnapkcs^−^* strain was significantly more sensitive to cisplatin than *adprt2^−^* cells (Fig. 5D). Taken together, these data reveal a role for NHEJ in the tolerance of cisplatin-induced DNA damage in the absence of Adprt2.

## DISCUSSION

Our previous work identified that the ARTs Adprt2 and Adprt1b are required for tolerance of *Dictyostelium* cells to DNA SSBs, whereas Adprt1a is required to promote repair of DSBs by NHEJ (Couto et al., 2011). Adprt1a-mediated repair of DSBs is regulated, in part, through a PBZ domain in *Dictyostelium* Ku70 that is required to enrich the protein at sites of DNA damage. This domain is unusually prevalent in *Dictyostelium*, being apparent in a greater number of proteins implicated in the DDR than in humans, suggesting that ADP-ribose interaction domains might act as surrogate markers for new DNA repair proteins (Ahel et al., 2008). Here, we identify *Dictyostelium* proteins that contain the ADP-ribose binding macrodomain and characterise APL as a protein enriched in chromatin in response to the cisplatin in a manner that this is dependent on its macrodomain and the ART Adprt2.

The macrodomain of APL has undergone a circular permutation. This mutation is apparent in all dictyostelids in which the genomes have been sequenced. This rearrangement is not unique to *Dictyostelium*, with a similar permutated macrodomain being present in *Arabidopsis thaliana* and *Oryza sativa*. Structural analysis of this novel macrodomain indicates it has retained the core features of this domain family, including the α-β-α sandwich fold consisting of a six-stranded β-sheet flanked by α-helices (Rack et al., 2016). Moreover, several key amino acids within the ADP-ribose-binding pocket that coordinate ADP-ribose binding are conserved in this domain. Most notably they retain an amino acid (D175 and E439) at an equivalent position to D723 of human ALC1 (Ahel et al., 2009; Gottschalk et al., 2009) and D20 of *Archaeoglobus fulgidus* AF1521 (Karras et al., 2005) that is crucial for ADP-ribose binding. Additionally, F113, which forms a binding pocket for the distal ribose unit, is absolutely conserved, and the two substrate-binding loops (loops 1 and 2) that flank the pyrophosphate of the ADP-ribose are also apparent (Ahel et al., 2009; Chen et al., 2011; Gottschalk et al., 2009; Karras et al., 2005). Consistent with the permutated macrodomain of *Dictyostelium* APL being able to interact with ADP-ribose, we observe that this domain is able to bind ADP-ribose polymers *in vitro* (Fig. 2D,E). Overall, these data indicate that although the macrodomain has undergone a circular permutation, it has retained its tertiary structure and its ability to interact with ADP-ribose.

APL contains several domains that suggest it plays a role in DNA repair. In addition to the macrodomain, it also contains a central PBZ domain. This motif is present in three human proteins, all of which are implicated in DNA repair (Ahel et al., 2008). Additionally, whereas the PBZ domain is more prevalent in *Dictyostelium*, all the proteins that contain this domain are implicated either in DNA repair directly (e.g. Ku70 and Ung), or the wider DDR (e.g. Rad17, Chk2 and CHFR). Additionally, APL also contains an FHA domain at its N-terminus, which is homologous to the FHA domain in other organisms that interact with the DNA repair proteins XRCC1 and XRCC4 (Ali et al., 2009; Bekker-Jensen et al., 2007; Iles et al., 2007; Loizou et al., 2004; Luo et al., 2004). These observations led us to speculate that APL might function in DNA repair. Consistent with this hypothesis, we observe that APL is enriched in chromatin following exposure of cells to cisplatin, an agent that induces DNA ICLs (Fig. 3A). Cisplatin is also able to induce DNA intra-strand crosslinks, primarily between neighbouring guanine nucleotides, raising the possibility that APL is detecting this variety of DNA damage, as opposed to ICLs (Eastman, 1983; Fichtinger-Schepman et al., 1985). Importantly, however, we do not observe enrichment of APL in chromatin following exposure of cells to agents that induce base damage repaired by base excision repair (BER, induced by MMS), or bulky DNA adducts that are repaired by nucleotide excision repair (NER; induced by 4-NQO; Fig. 3A). Therefore, we believe that APL is responding to DNA ICLs, as opposed to other varieties of DNA damage induced by cisplatin.

Sequence analysis reveals no obvious motifs in APL that might perform a catalytic role in the processing or repair of DNA damage. Although a proportion of macrodomains are known to remove ADP-ribose moieties from proteins, as opposed to binding ADP-ribosylated proteins (Barkauskaite et al., 2015; Jankevicius et al., 2013; Rosenthal et al., 2013; Sharifi et al., 2013; Slade et al., 2011), we have been unable to detect any such activity in APL (data not shown). Taken together, these data suggest a more structural role for APL in sensing signals induced by DNA ICLs, rather than direct modulation of DNA lesions. In this regard, the overall domain architecture of APL is similar to APLF, a vertebrate protein implicated in promoting DNA strand break repair by facilitating accumulation of repair proteins at damage sites (Bekker-Jensen et al., 2007; Iles et al., 2007; Kanno et al., 2007; Rulten et al., 2008, 2011). Although both proteins contain an N-terminal FHA domain and central PBZ domain, the C-terminal PBZ domain of APLF has been replaced by a macrodomain in APL. Macrodomains have been proposed to bind terminal ADP-ribose moieties within PAR chains (Karras et al., 2005), whereas PBZ domains bind the ADP-ribose–ADP-ribose junction and adenine rings internal to ADP-ribose polymers (Eustermann et al., 2010; Li et al., 2010; Oberoi et al., 2010). It is interesting to speculate, therefore, that the macrodomain and PBZ domain of APL might act in tandem to bind internally to the PAR chain and the terminal ADP-ribose unit respectively to facilitate high-affinity binding to ADP-ribose polymers.

The occurrence of APL-like macrodomains in very distant organisms, such as *Dictyostelium* species and plants, suggests a general utility of this module to support DNA repair signalling. Of note, the APL macrodomain in plants is fused to two other DNA repair domains, aprataxin and polynucleotide kinase domains (Rack et al., 2016), strongly implying that APL supports DNA repair in plants as well. Furthermore, as in vertebrate aprataxin, PNK as well as APLF are FHA-domain-containing proteins that interact with DNA repair ligase complexes. Given that the APLF domain structure is not preserved in plants and Dictyostelids (Mehrotra et al., 2011), it is tempting to speculate that APL might be supporting the PAR-binding function instead of APLF in these organisms.

Our data indicate a hitherto unrecognised role for ADP-ribosylation in the cellular response to cisplatin, an agent that induces DNA ICLs. This is based on our observations that (1) substantial nuclear ADP-ribosylation is observed in response to the ICL-inducing agent cisplatin, (2) enrichment of APL in chromatin in response to cisplatin is dependent on its macrodomain and the ART Adprt2, and (3) the *adprt2^−^* strain is sensitive to cisplatin. Our data in *Dictyostelium* (Couto et al., 2013, 2011; Pears et al., 2012), in addition to those of others in vertebrates (Gibson and Kraus, 2012), implicate ARTs in repair of SSBs and DSBs. Therefore, it is possible that ARTs are detecting these or similar DNA architectures following processing of cisplatin-induced DNA damage, rather than the ICL directly. However, although Adprt2 is required for tolerance to DNA SSBs, the enrichment of APL in chromatin, an event that is dependent on Adprt2, does not occur in response to canonical base damage induced by MMS or 4-NQO. Similarly, no gross enrichment of APL is observed in chromatin following DNA DSBs, and the Adprt2-null strain is not sensitive to agents that induce this variety of DNA damage (Couto et al., 2013). Therefore, we believe Adprt2-mediated ADP-ribosylation induced by cisplatin is not induced by these DNA damage types directly, or if so, it is in the context of these DNA structures being produced as a consequence of DNA ICL processing.

Resolution of ICLs is facilitated by combining a number of repair pathways. In prokaryotes and lower eukaryotes such as budding yeast, repair is initiated by the NER apparatus that incises adjacent to the ICL to ‘unhook’ the lesion. The Pso2 nuclease digests past the unhooked ICL, producing a gapped intermediate that is filled-in by translesion synthesis (TLS) using low-fidelity DNA polymerases. The remaining crosslinked strand is removed by homologous recombination or a second round of NER (Dronkert and Kanaar, 2001; Lehoczky et al., 2007; Sengerova et al., 2011). Although a similar pathway has been proposed in mammalian cells (Ben-Yehoyada et al., 2009; Muniandy et al., 2009; Smeaton et al., 2008), the principal mechanism for ICL repair occurs during S-phase and is coordinated by the Fanconi anaemia pathway (Kottemann and Smogorzewska, 2013). ICLs result in stalling of replication forks that are detected by the Fanconi anaemia core complex. The FANCL component of this complex ubiquitylates FANCD2 and FANCI, which serves as a platform to coordinate a number of downstream factors. These include the nuclease FAN1 and SLX4, which acts as a scaffold for other nucleases including XPF (also known as ERCC4), Mus81 and SLX1. Following incision either side of the ICL on one DNA strand, in addition to potential processing by Pso2 (SNM1A), TLS bypasses the lesion. If replication forks have converged on the ICL, this process results in a DSB that is repaired by homologous recombination. In the absence of replication fork convergence, the remaining ICL is either removed by NER, or TLS results in a one-sided DSB that is resolved by homologous recombination (Sengerova et al., 2011).

*Dictyostelium* shares the core components of all pathways implicated in repair of ICLs, including the Fanconi anaemia pathway (dictybase.org) (Hsu et al., 2011; Lee et al., 1998, 1997; McVey, 2010; Yu et al., 1998; Zhang et al., 2009). It is possible that Adprt2 could be acting at any stage of these pathways. For example, it could directly detect DNA ICLs, either during S-phase or another stage of the cell cycle. Alternatively, as alluded to above, it could signal other DNA architectures resulting from processing of ICLs, most notably gapped single-stranded DNA intermediates and/or DNA DSBs. In this regard, Adprt2 has analogous functions to vertebrate PARP1, being required for tolerance to DNA SSBs, but playing a minor role in promoting NHEJ (Couto et al., 2011). Given that PARP1 has also been implicated in promoting the re-start of damaged or stalled replication forks (Bryant et al., 2009; Sugimura et al., 2008; Yang et al., 2004), it is interesting to speculate that Adprt2 and ADP-ribosylation might be acting in a similar pathway, although in the context of repairing damaged replication forks that encounter DNA ICLs. It should be noted, however, that during vegetative cell growth *Dictyostelium* cells have no discernible G1, with ∼10% of cells undergoing DNA replication, and the majority being in the G2 phase of the cell cycle (Couto et al., 2013; Muramoto and Chubb, 2008; Weijer et al., 1984). Given that up to 80% of cells display ADP-ribosylation foci following cisplatin treatment (Fig. 4B), this might indicate an S-phase-independent role for Adprt2-mediated ADP-ribosylation in DNA ICL repair. In this regard, *Dictyostelium* Fanconi anaemia mutants display only mild sensitivity to ICLs, whereas an *xpf*^−^ strain is extremely sensitive to this variety of DNA damage (Zhang et al., 2009). Furthermore, ADP-ribosylation has previously been implicated in resolution of UV-induced DNA damage by NER, a pathway that acts independently of S-phase (Fischer et al., 2014; Pines et al., 2012; Robu et al., 2013). It will therefore be interesting to more formally assess whether Adprt2 functions in conjunction with the Fanconi anaemia pathway during DNA replication, or might be involved in an excision repair pathway at other stages of the cell cycle.

Although *adprt2^−^* cells display sensitivity to cisplatin, substantial nuclear ADP-ribosylation is evident in these cells and is dependent on Adprt1a. Taken together, these data indicate that although Adprt2 is required for tolerance to cisplatin, in its absence Adprt1a can signal DNA damage to maintain cell viability in the face of DNA damage. Redundancy exists between ARTs in signalling DNA damage. For example, PARP1 and PARP2 both respond to DNA base damage, and redundancy between these ARTs has been implied by the embryonic lethality of *Parp1^−/−^Parp2^−/−^* mice (Menissier de Murcia et al., 2003). Moreover, PARP1 and PARP3, the functional orthologues of *Dictyostelium* Adprt2 and Adprt1a respectively, act synergistically in response to ionising radiation in mouse and human cells (Boehler et al., 2011). Our observations of redundancy between Adprt2 and Adprt1a in signalling cisplatin-induced DNA damage is reminiscent of the situation in signalling DNA base damage in *Dictyostelium*. In the absence of Adprt2, SSBs are converted into DSBs that are subsequently signalled by Adprt1a to promote NHEJ (Couto et al., 2013). Consistent with a similar scenario occurring in response to cisplatin, we observe that disruption of NHEJ in combination with Adprt2 also further sensitises cells to cisplatin, indicating that NHEJ is a functional pathway in ICL repair in *Dictyostelium* providing tolerance of these lesions in the absence of Adprt2. Although a defective Fanconi anaemia pathway can channel repair through NHEJ the impact on cell viability is variable depending on the organism studied, or the NHEJ components disrupted. For example, disruption of the NHEJ pathway in *C. elegans* and humans supresses the sensitivity of Fanconi anaemia mutants to ICLs (Adamo et al., 2010). A similar reversal of ICL sensitivity is also observed in Fanconi-anaemia-defective chicken DT40 cells when disrupting *Ku70*, although this is not the case when disrupting other NHEJ factors, such as DNA-PKcs or ligase IV (Pace et al., 2010). In contrast, experiments using mouse embryonic fibroblasts indicate that disruption of *Fancd2* and *Ku80* or *53bp1* in combination increases genome instability and sensitivity to ICLs (Bunting et al., 2012; Houghtaling et al., 2005). Our data indicating that disruption of Adprt2 and NHEJ in combination further sensitises cells to cisplatin suggests that, similar to the studies in mice, in the absence of effective ICL repair NHEJ performs a beneficial role in allowing cells to tolerate agents such as cisplatin. One potential explanation for these data is the cell cycle distribution of vegetative *Dictyostelium* cells*.* For example, NHEJ is generally toxic during S-phase, whereas it is effectively utilised in G2 (Beucher et al., 2009; Rothkamm et al., 2003). Given that the majority of *Dictyostelium* cells are in G2 during vegetative cell growth, it is conceivable that loss of effective ICL repair and subsequent engagement of NHEJ is beneficial in this stage of the cell cycle.

In summary, our search for new macrodomain-containing proteins identified APL as a factor that is able to interact with ADP-ribose polymers *in vitro*. The presence of FHA and macrodomains in this protein implicate it in the cellular response to DNA damage and, consistent with this hypothesis, we observe that APL is enriched in chromatin specifically in response to an agent that induces DNA ICLs. The dependence of this event on the macrodomain of APL implicates ADP-ribosylation in this response, and, consistent with this hypothesis, we find that the ART Adprt2 is required to ADP-ribosylate proteins in response to cisplatin exposure. Furthermore, in the absence of Adprt2, we uncover a role for NHEJ in allowing cells to tolerate cisplatin. Taken together, these data illustrate redundancy between ARTs that signal alternative varieties of DNA damage to maintain cell viability in the face of genotoxic stress.

## MATERIALS AND METHODS

### Homology searching and multiple sequence alignments

*In silico* searches were performed within dictyBase (www.dictybase.org) and the non-redundant UniRef50 database (Basu et al., 2015; Wu et al., 2006). Proteins containing known macrodomains were identified in the Pfam and SUPERFAMILY protein databases (Finn et al., 2016; Wilson et al., 2009). Initial local similarity searches were formed using BLAST (Altschul et al., 1997). Profile hidden Markov models (profile-HMMs) were generated using HMMer2 and HMMer3, which were also used for profile-sequence homology searches, which were iterated up to 40 times (Eddy, 1996; Finn et al., 2011). HHpred was employed for profile–profile homology searches (Soding et al., 2005). Secondary structure predictions were performed with PsiPred (Jones, 1999).

Protein amino acid sequences were obtained from UniProt or dictyBase (Basu et al., 2015; Wu et al., 2006). Alignments of protein sequences were performed using MUSCLE or T-Coffee, and visualised in Belvu (Edgar, 2004; Notredame et al., 2000; Sonnhammer and Hollich, 2005). DNA sequences were aligned using the MultAlin interface (Corpet, 1988).

### Protein expression and purification

GST-tagged proteins were generated by amplifying the following regions of the *apl* gene from cDNA and ligation into pGEX-4T-1 (GE Healthcare): GST–APL (nucleotides 1–1689), GST–FHA (nucleotides 1–336), GST–PBZ (nucleotides 504–591), GST–MACRO (nucleotides 1026–1689). GST-tagged proteins were expressed and purified according to the manufacturer's instructions. A selenomethionine-substituted *D. discoideum* macrodomain protein was produced with SelenoMet Medium Base and Nutrient Mix (Molecular Dimensions) as per the manufacturer's instructions and purified as above.

### Crystallisation, data collection and processing

Crystallisation trials were performed with proteins at 25 mg/ml in buffer containing 150 mM NaCl, 1 mM DTT and 25 mM Tris-HCl pH 7.5, at 20°C with commercial screens using the sitting-drop vapour-diffusion method. Crystallisation drops were set up with the aid of a Mosquito Crystal robot (TTP Labtech) using 200 nl of protein solution plus 200 nl of reservoir solution in MRC two-well crystallisation microplates (Swissci) equilibrated against 75 µl of reservoir solution. Co-crystallisation trials were set up by adding 2 mM ADPr to the protein for at least 1 h prior to setting up crystallisation drops. Crystals of the macrodomain proteins were grown in 0.2 M lithium sulphate, 0.1 M phosphate/citrate and 20% (w/v) PEG1000 (*Dictyostelium*), and in 0.1 M SPG buffer, pH 4 (succinic acid, sodium phosphate monobasic monohydrate and glycine) and 25% (w/v) PEG 1500 (*O. sativa*). Crystals were cryoprotected by transfer into reservoir solution before being vitrified by submersion in liquid nitrogen. X-ray data were collected at beamlines I04 of the Diamond Light Source (Rutherford Appleton Laboratory, Harwell, UK) and data collection statistics are shown in Table S1. X-ray data were processed using Xia2 (Winter et al., 2013). *Dictyostelium* macrodomain X-ray data was phased with AUTOSOL (Terwilliger et al., 2009). PHASER (Storoni et al., 2004) was used to solve the *O. sativa* macrodomain data by molecular replacement with the *Dictyostelium* macrodomain structure. Model building for all structures was carried out with COOT (Emsley and Cowtan, 2004) and real space refinement with REFMAC5 (Murshudov et al., 1997), coupled with automatically generated local non-crystallographic symmetry restraints. Structural figures were prepared using PyMOL (Molecular Graphics System, Version 1.3 Schrödinger, LLC).

### PAR-binding assays

GST-tagged proteins were serially diluted and increasing concentrations between 0.625 pmol to 2.5 pmol of proteins were either slot-blotted or dot-blotted onto a nitrocellulose. The membrane was blocked with 5% milk in Tris-buffered saline with 0.05% Tween-20 (TBST), before incubation with PAR polymers (Trevigen). The membrane was then washed with TBS-T, followed by four washes with TBS-T with 1 M NaCl, and a further wash with TBS-T. Detection was performed by western blotting with anti-PAR (1:1000; cat. no. 4336-BPC-100, Trevigen) and anti-GST (1:3000; cat. no. G7781, Sigma-Aldrich) antibodies.

### Cell culture and strain generation

*Dictyostelium* cells were grown according to standard procedures, either axenically or on SM agar plates in association with *Klebsiella aerogenes*. Generation of the *adprt1a^−^*, *adprt2^−^*, *adprt1a^−^ adprt2^−^* double mutants, *dnapkcs^−^*, *dnapkcs^−^ adprt2^−^* double mutants and *dclre1^−^* cells was as previously described (Couto et al., 2013, 2011; Hudson et al., 2005). To generate an *apl^−^* strain, DNA fragments upstream (nucleotides −1031 to −3, relative to the transcription start site) and downstream (nucleotides +1916 to +2849) of the *apl* gene were amplified by PCR and ligated into the pLPBLP vector (dictyBase) to flank a blasticidin-resistance cassette (Faix et al., 2004). The disruption construct was excised from the pLPBLP vector by restriction digestion with HpaI and NotI, and was transfected into Ax2 cells using standard procedures. Blasticidin was added the following day at a concentration of 10 μg/ml to provide selection. Blasticidin-resistant clones were isolated and screened for *apl* disruption by PCR and Southern blotting (Fig. S2).

To express Myc–APL in *Dictyostelium* strains, the cDNA sequence of full-length APL or Myc–APL-Δ342-563 was amplified by PCR, utilising primers to introduce an in-frame N-terminal Myc-tag, and ligated into pDXA-3C (dictyBase). Plasmids were electroporated into *Dictyostelium* cells alongside the pREP helper plasmid (dictyBase) according to standard procedures. Cells expressing Myc–APL were selected for by addition of 10 μg/ml G418 (Sigma-Aldrich) after 24 h.

### Subcellular fractionation

Exponentially growing *Dictyostelium* cells were resuspended to a density of 5×10^6^ cells/ml in HL5 and incubated with genotoxic agents (Sigma-Aldrich). For MMS, phleomycin and 4-NQO, incubation was for 1 h (4-NQO-treated cells were incubated in the dark). For cisplatin, cells were resuspended to 5×10^6^ cells/ml in Pt buffer (1 mM NaPO_4_, 3 mM NaCl, pH 6.5) and incubated in the dark for 5 h. Following incubation, the cells were washed with KK2 and resuspended in nuclear lysis buffer [50 mM HEPES, pH 7.5, 150 mM NaCl, 1 mM EDTA, phosphatase inhibitor cocktail 2 and 3 (Sigma-Aldrich), proteasome inhibitor cocktail (Roche), 10 mM benzamide (Sigma-Aldrich), 200 μM DEA (Trevigen)] with 0.1% Triton X-100 to a density of 5×10^6^ cells/ml. Cells were incubated on ice for 15 min, before centrifugation at 14,000 ***g*** for 3 min at 4°C. The pellet was resuspended in the same volume of nuclear lysis buffer with 0.1% Triton X-100, and incubated on ice for 15 min, before centrifugation at 14,000 ***g*** for 3 min at 4°C. The pellet was resuspended in nuclear lysis buffer with 200 μg/ml RNase A (Sigma-Aldrich), and incubated for 30 min at room temperature with rotation, before centrifugation as above. The final pellet was resuspended in 2× SDS loading buffer containing 100 μM DTT prior to boiling for 5 min. Whole-cell extracts were prepared by washing cells in KK2, and resuspending in 2× SDS loading buffer containing 100 μM DTT, prior to boiling for 5 min.

Analysis of extracts was performed by SDS-PAGE and western blotting with the following primary antibodies: anti-Myc (1:1000; cat. no. sc-40, Santa Cruz Biotechnology), anti-H3 (1:2000; cat. no. ab12079, Abcam), anti-γH2AX (1:1000; cat. no. ab11174, Abcam), anti-actin (1:1000; cat. no. sc-1615, Santa Cruz Biotechnology) and anti-pan-ADP-ribose binding reagent (1:1000; MABE1016, Millipore).

### Immunofluorescence

Exponentially growing *Dictyostelium* cells were resuspended to a density of 10^6^ cells/ml in HL5 and allowed to adhere to glass coverslips for 30 min. The HL5 was then removed and the coverslips washed with Pt buffer. Cells were then exposed to 300 μM cisplatin for the indicated times, in the dark. Coverslips were incubated for 5 min in ice-cold nuclear extraction buffer (10 mM PIPES, pH 6.8, 300 mM sucrose, 3 mM MgCl_2_, 20 mM NaCl, 0.5% Triton X-100) and washed twice with TBS. Cells were fixed with ice-cold 70% ethanol for 5 min, followed by the addition and immediate removal of ice-cold 100% methanol, prior to washing three times with TBS.

Coverslips were blocked with 3% BSA in TBS for 1 h, prior to a 2-h incubation with an anti-pan-ADP-ribose binding reagent (MABE1016; Millipore) in 3% BSA. Coverslips were washed three times in TBS, then incubated in the dark for 1 h with a TRIT-C-conjugated anti-rabbit-IgG secondary antibody (R0156; Dako), followed by three further TBS washes. Coverslips were mounted onto glass slides using VECTASHIELD mounting medium containing DAPI (Vector Laboratories) and visualised with a microscope (1×71; Olympus). 250 nuclei were analysed per condition. Images were acquired on a camera using HCImage Acquisition (Hamamatsu Photonics) image software and processed in Photoshop (Adobe).

### DNA damage survival assays

Exponentially growing *Dictyostelium* cells were resuspended to 10^6^ cells/ml in Pt buffer, and exposed to the indicated concentrations of cisplatin (Sigma-Aldrich). Cells were incubated in shaking suspension at 100 rpm for 5 h in the dark. 10^4^ cells were diluted 1:100 in KK2 and 250 cells mixed with 350 μl *K. aerogenes* and transferred to 140 mm SM agar plates in duplicate. The plates were incubated in the dark and survival assessed by observing plaque formation after 3, 4, 5 and 6 days.
